# Role of *Helicobacter pylori* and Other Environmental Factors in the Development of Gastric Dysbiosis

**DOI:** 10.3390/pathogens10091203

**Published:** 2021-09-16

**Authors:** Uriel Gomez-Ramirez, Pedro Valencia-Mayoral, Sandra Mendoza-Elizalde, Juan Rafael Murillo-Eliosa, Fortino Solórzano Santos, Araceli Contreras-Rodríguez, Gerardo Zúñiga, Pamela Aguilar-Rodea, Verónica Leticia Jiménez-Rojas, Juan Carlos Vigueras Galindo, Marcela Salazar-García, Norma Velázquez-Guadarrama

**Affiliations:** 1 Laboratorio de Investigación en Enfermedades Infecciosas, Hospital Infantil de México Federico Gómez, Mexico City 06720, Mexico; urielgoramirez93@outlook.es (U.G.-R.); hipsme@hotmail.com (S.M.-E.); solorzanof056@gmail.com (F.S.S.); qbp_pam@hotmail.com (P.A.-R.); verozenemij@hotmail.com (V.L.J.-R.); jcvazul@yahoo.com (J.C.V.G.); 2 Posgrado en Ciencias Quimicobiológicas, Escuela Nacional de Ciencias Biológicas, Instituto Politécnico Nacional, Mexico City 11340, Mexico; 3 Departamento de Patología Clínica y Experimental, Hospital Infantil de México Federico Gómez, Mexico City 06720, Mexico; vamp_48@yahoo.com (P.V.-M.); ralfdd_67@hotmail.es (J.R.M.-E.); 4 Departamento de Microbiología, Escuela Nacional de Ciencias Biológicas, Instituto Politécnico Nacional, Mexico City 11340, Mexico; aracelicontreras21@gmail.com; 5 Laboratorio de Variación Biológica y Evolución, Departamento de Zoología, Escuela Nacional de Ciencias Biológicas, Instituto Politécnico Nacional, Mexico City 11340, Mexico; capotezu@hotmail.com; 6 Laboratorio de Investigación en Biología del Desarrollo y Teratogénesis Experimental, Hospital Infantil de México Federico Gómez, Mexico City 06720, Mexico; msalazar.investigacion@gmail.com

**Keywords:** gastric microbiota, *Helicobacter pylori*, non-*Helicobacter pylori* bacteria, eradication therapy, dysbiosis, probiotics

## Abstract

Microbiomes are defined as complex microbial communities, which are mainly composed of bacteria, fungi, and viruses residing in diverse regions of the human body. The human stomach consists of a unique and heterogeneous habitat of microbial communities owing to its anatomical and functional characteristics, that allow the optimal growth of characteristic bacteria in this environment. Gastric dysbiosis, which is defined as compositional and functional alterations of the gastric microbiota, can be induced by multiple environmental factors, such as age, diet, multiple antibiotic therapies, proton pump inhibitor abuse, *H. pylori* status, among others. Although *H. pylori* colonization has been reported across the world, chronic *H. pylori* infection may lead to serious consequences; therefore, the infection must be treated. Multiple antibiotic therapy improvements are not always successful because of the lack of adherence to the prescribed antibiotic treatment. However, the abuse of eradication treatments can generate gastric dysbiotic states. Dysbiosis of the gastric microenvironment induces microbial resilience, due to the loss of relevant commensal bacteria and simultaneous colonization by other pathobiont bacteria, which can generate metabolic and physiological changes or even initiate and develop other gastric disorders by non-*H. pylori* bacteria. This systematic review opens a discussion on the effects of multiple environmental factors on gastric microbial communities.

## 1. Introduction

The human microbiome is a collective of pathogenic and commensal microbial communities that colonize the human body [1]. These communities significantly influence human physiology in processes related to energy homeostasis, neurodevelopment, nutrition, immunologic activity, resistance to pathogens, and gut epithelial health [2,3]. It has been estimated that each person is colonized by a microbial density of approximately 10^14^ cells, showing that the number of microbial cells exceeds the total number of cells present in the human body [4]. In the stomach, it is assumed that a specific microbial composition coexists that is different from the established microbiota in other segments of the gastrointestinal tract [5], due to its unique compartmentalization, providing an acidic microenvironment in this organ, and inhibiting the growth of most opportunistic bacteria. These microenvironmental conditions allow only a few bacteria to optimally colonize the gastric epithelium [6], e.g., *Helicobacter pylori.*

Since the discovery of *H. pylori* in 1982, much has been uncovered about this microorganism, specifically relating to its association with several gastric diseases, both in the initiation and development [7]. The persistence of *H. pylori* in the stomach is usually associated with the development of gastroduodenal diseases [8]. However, this bacterium has colonized most of the world’s population, with most cases being asymptomatic [8]. The increasing rates of employment and the abuse of unnecessary antibiotic therapies for disease prevention or treatment have raised the question of the possible alterations in the native gastric microbiome and its consequences on human health. The purpose of this review is to update the awareness about the roles of *H. pylori*, non-*H. pylori* bacteria, and multiple environmental factors in the development of dysbiotic states in the gastric mucosa, and its related consequent disorders.

## 2. Methodology

### 2.1. Study Design

In this review, we attempted to assess the roles of *H. pylori*, non-*H. pylori* bacteria, and other environmental factors on the development of gastric dysbiosis, and its consequent gastric disorders. The present study was conducted using available data from worldwide published reports. Past studies were previously classified based on the selection of the study subjects by *H. pylori* status, disease status of the subjects (i.e., whether healthy subjects or patients diagnosed with gastric diseases caused by *H. pylori* infection), body site in the study (i.e., stomach), and the methodologies employed in the studies (such as culture-dependent methods, pyrosequencing, RT-PCR, and next-generation sequencing technologies). In this work, a special focus was given to *H. pylori*-positive and negative patients who developed gastric disorders probably caused by non-*H. pylori* microbiota.

### 2.2. Article Research

A review of several full-text articles and reviews was performed through two main databases (i.e., PubMed and Google Scholar), from February 2020 to July 2021 on the effects of *H. pylori*, non-*H. pylori* bacteria, and environmental factors in the gastric microbiota of patients and healthy subjects. This study identified 233 records after removing duplicates (*n* = 1473). After screening through titles and abstracts, 148 records were retained and assessed for eligibility.

### 2.3. Selection of Studies

Past studies that were previously identified in the literature were explored by their title and abstract. Articles and reviews with relevant abstracts were closely examined. The inclusion and exclusion criteria of the studies were determined by the investigators before the revision of the selected literature. The inclusion criteria were as follows: (1) original full-text articles that provided reproducible and sufficient original data about *H. pylori* colonization and/or infection and the employment of antibiotic treatments for the eradication of the infection; (2) original full-text articles that evaluated the effect of environmental factors, such as antibiotic therapies for bacterial infection eradication on the gastric microbiota of patients with different gastric disorders (including chronic gastritis, intestinal metaplasia, and gastric cancer), and healthy subjects; (3) original full-text articles employing methodologies included culture-dependent methods, pyrosequencing, RT-PCR, and next-generation sequencing technologies; (4) original full-text articles in English, and (5) reviews that involved human gastric metagenomic data, classified by the host health status. The articles that did not fulfil these criteria were excluded from this review.

### 2.4. Data Extraction

**Figure 1:**
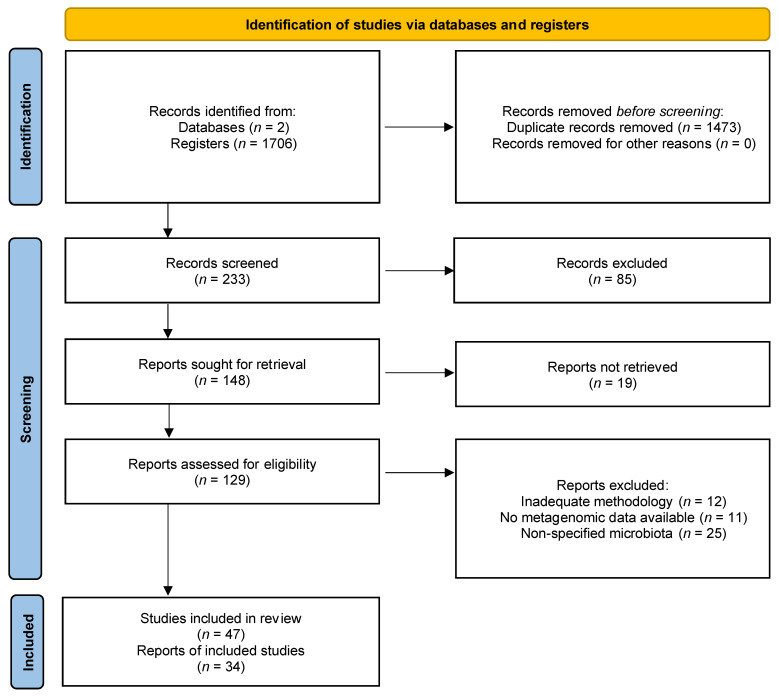
PRISMA flow diagram for the identification and selection of articles on the effects of *H. pylori*, non-*H. pylori* bacteria, and multiple environmental factors on the gastric microbiota of patients and healthy subjects [9].

The authors confirmed the characteristics of each selected study, including title, first author’s surname, abstract, study subject conditions, *H. pylori* status, materials and methods, results, and the data obtained from stomach metagenomic analyses (Figure 1).

## 3. Main Findings and Discussion

### 3.1. Gastrointestinal Microbiota

The evolution of *Homo sapiens sapiens* has been irrevocably linked to the mutualistic relationships within the gastrointestinal microbiota [10]. Several methodologies have been used to identify the main genera residing in the gastrointestinal microbiota of healthy individuals such as culture-dependent methods; moreover, the employment of recent molecular technologies such as next-generation sequencing technologies has enabled the identification of more than 120 dominant phylotypes [1,11,12].

The human gastrointestinal microbiota contains a vast majority of bacterial species that belong to main phyla such as Bacteroidetes, Firmicutes, Verrucomicrobia, Proteobacteria, Actinobacteria, and Fusobacteria [13]. A study performed by Mailhe et al. included biopsy results obtained by endoscopy and colonoscopy procedures from the stomach (pH 1.8), duodenum (pH 2.5), ileum (pH 6.6), right (pH 6.9), and left (pH 7.1) colon. The study revealed relative abundances in the lower gastrointestinal microbiota than the microbiota present in the stomach and duodenum. Five main genera (*Streptococcus*, *Escherichia*, *Bacteroides*, *Lachnoclostridium*, and *Blautia*) and three species (*Streptococcus salivarius*, *Escherichia coli*, and *Bacteroides uniformis*) were identified and found colonizing different parts of the gastrointestinal tract [14]. Microbial composition and its abundance can significantly vary with diet and exposure to different compounds (e.g., drugs and pH), for instance, the acidic environment in the stomach (pH = 2–4) [15].

### 3.2. Gastric Microbiota

Microenvironmental characteristics such as pH, mucus density, bile, and peristaltic movements make the stomach a complex organ. This organ presents anatomical and histological characteristics, which are suitable for a specific and different microbial composition as compared to the other segments of the gastrointestinal tract [6]. The commensal and pathogenic bacterial growth is usually controlled by the prevailing conditions in the gastric region, due to its unique and heterogeneous habitat that harbors a specific number of microorganisms [6]. Predominant phyla in the stomach mainly include Proteobacteria, Firmicutes, Actinobacteria, Fusobacteria, and Bacteroidetes. Among these, the most abundant class is Bacilli (order Lactobacillales), the most prevailing family is *Streptococcaceae* (genus *Streptococcus*), the most prevailing genera are *Prevotella*, *Rothia*, *Fusobacterium*, and *Veillonella* (in decreasing order) [16]. Dekaboruah et al. reported that genera *Neisseria*, *Haemophilus*, *Porphyromonas*, and family *Pasteurellaceae* constituted approximately 70.5% of the total gastric microbiota [17].

The role of the gastric microbiota is mainly associated with metabolic functions, such as degradation of undigested carbohydrates and unabsorbed molecules, for the regulation of gastrointestinal homeostasis via the synthesis of short-chain fatty acids (SCFs) by gastrointestinal epithelial cells; control of the proliferation and differentiation of epithelial cells in addition to the development and maintenance of homeostasis by SCFs; maturation of the immune system through the development and regulation of the immune system and prevention of inappropriate inflammation development; energy balance, vulnerability to diseases (competition by the production of bacteriocins and inhibition of attachment and further invasion of pathogenic bacterial cells), and specific host behaviours [16,17], all of which contribute to the maintenance of a healthy microbiota.

### 3.3. Dysbiosis Generated in the Gastric Microenvironment by Helicobacter pylori Status Diseases

#### 3.3.1. Dysbiosis

**Figure 2:**
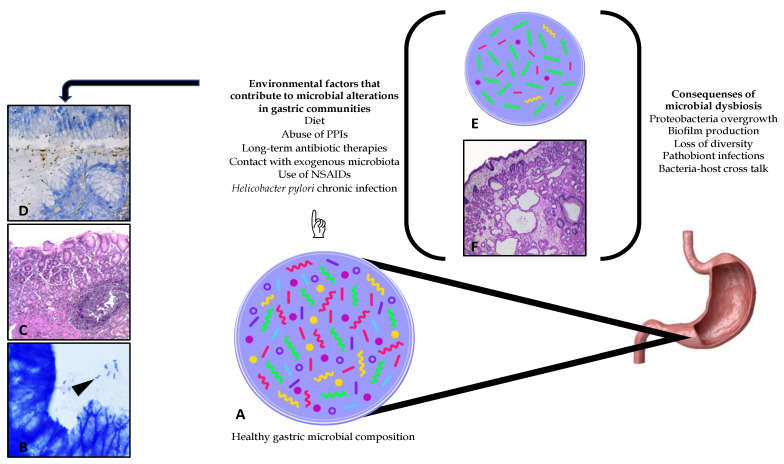
Schematic of the gastric microbial composition and alterations generated by multiple environmental factors. (**A**) Indicates a schematic of a healthy gastric microbiota. A healthy microbiota is defined as the coexistence of microbial communities, such as pathogenic and symbiotic bacteria with no harmful consequences. Nevertheless, dysbiotic communities can be generated due to several environmental factors, especially by *Helicobacter pylori* chronic infection, which can be diagnosed through histopathological analysis and differential staining techniques; (**B**) Giemsa stain, 100×. Arrow highlight *H. pylori* in biopsy; (**C**) H&E stain, 40×; (**D**) anti-Hp stain, 100×. The presence of dysbiotic communities in the gastric microenvironment usually favours the continuous loss of bacterial diversity; (**E**) increasing infections caused by pathobionts and the growth of antibiotic-resistant gene bacteria. Histological damage can also be observed, caused by environmental factors, that is, the abuse of PPIs (**F**).

A healthy microbiota is defined as the coexistence of microbial communities, such as pathobionts and commensal bacteria with no harmful consequences. However, a person’s lifestyle and habits may lead to several significant alterations in the native composition and structure of the gastrointestinal microbiota [2]. In addition, the composition of native gastric microbiota is affected by several factors, such as host health status, *H. pylori* colonization, chronic infection and subsequent complications, the use and abuse of drugs and toxins, age, surgical interventions, inflammation, alcoholism, smoking, use of nonsteroidal anti-inflammatory drugs (NSAIDs), use of proton pump inhibitors (PPIs), and others [18], as shown in Figure 2. These conditions generate dysbiosis, defined as compositional and functional alterations of the microbiota of the host.

#### 3.3.2. *Helicobacter pylori*

Since the discovery of *Helicobacter pylori* in the 20th century, metagenomic studies have focused on elucidating the role of *H. pylori* in the gastric microbiota of healthy subjects as well as on its role in the development of gastric disorders of infected patients [19,20]. There are some concepts related to the coevolution of *H. pylori* and its intimate association with humans that has enabled this bacterium to develop survival strategies and persist in the stomach. *H. pylori* is considered a member of the gastric and oral microbiota, with functions that are not yet fully understood [21,22]. Some studies suggest that the presence of this microbe may be beneficial to some people by reducing the risk of developing asthma during childhood; decreasing the frequency of inflammatory bowel and celiac diseases; interfering with obesity, among other issues [23].

Although it is estimated that 50% of the world’s population is colonized with *H. pylori*, only 1–2% develop several degrees of histological proinflammatory responses and evident complications of the gastric mucosa. Those responses have been attributed to host–environmental factors, such as continuous changes in the gastric pH, chronicity of the infection, specificity of the strain, genetic susceptibility of the host, hyperglycaemia, smoking, diet, and contact with exogenous microbiota (Figure 2) [24].

The interaction between virulent *H. pylori* strains, genetic background (*cagA* and *vacA H. pylori* genotypes), and host–environmental factors confined to the host, may influence the initiation and development of gastric diseases [22]. However, this bacterium is currently recognized as the main cause of peptic ulcers, chronic gastritis, and gastric adenocarcinoma, which represents 15.4% of cancers induced by infectious agents, and is the second leading cause of worldwide deaths related to cancer [25]. Maldonado-Contreras et al. found that members of the phylum Acidobacteria (class Acidobacteria), Proteobacteria (classes Alphaproteobacteria, Deltaproteobacteria, and Epsilonproteobacteria), and Spirochaetae (class Spirochetes) were correlated with the presence of *H. pylori* in infected patients [26]. By contrast, relative abundances of families *Bradyhizobiaceae*, *Caulobacteraceae*, *Lactobacillaceae*, and *Burkholderiaceae* were found to be significantly higher in *H. pylori*-negative patients when compared to *H. pylori*-positive patients [27].

#### 3.3.3. Gastric Dysbiosis in Patients with Gastritis and *H. pylori* Variable Status

During childhood, infection with *H. pylori* usually begins with simple chronic mononuclear inflammation that progresses into varying degrees of acute neutrophilic inflammation; because of the progression of inflammation, gastritis evolves to atrophic gastritis over the years, which is characterized by the loss of normal mucosa glands in the body and/or antrum. Thus, chronic inflammation is intimately associated with neutrophilic inflammation and cytotoxicity of the infective *H. pylori* strains [28].

**Figure 3:**
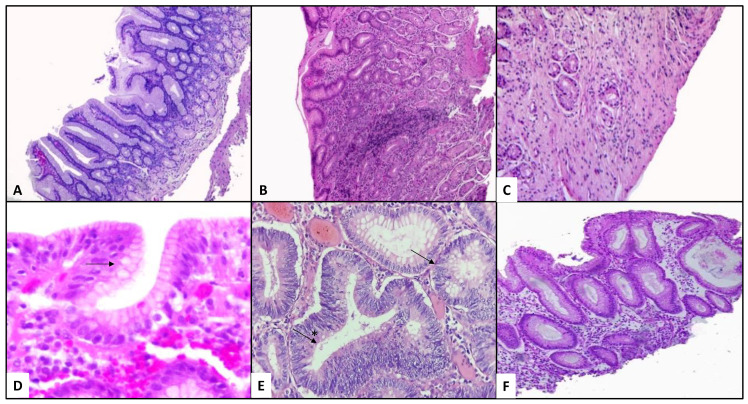
Histopathological alterations generated by *Helicobacter pylori* chronic infection. (**A**), near-normal antral mucosa is shown (H&E 10×). (**B**) illustrates a case of non-atrophic gastritis; increased mononuclear infiltrate with preserved antral architecture can be observed (H&E 10×). Loss of glands and increased connective tissue can be seen in (**C**), representing a case of atrophic gastritis (H&E 20×). (**D**) Goblet cells (arrow) are present in the foveolar epithelium which correspond to an initial lesion of intestinal metaplasia (H&E 40×). (**E**) Hyperchromatic nuclei with loss of its polarity corresponding to dysplasia in a gastric gland, are marked with a black arrow; complete distortion of a gland with cell proliferation and loss of its basal membrane are shown with a (*) marked arrow in a case of gastric adenocarcinoma (H&E 20×). (**F**) Mucosal distortion of the gastric antrum, with dilated and irregular glands, is observed from a patient treated with proton pump inhibitors (H&E 10×).

Gastritis is defined as an inflammatory condition of the gastric mucosa that shows variable symptoms in different patients, which depends on the response presented by the host) [29,30]. Most acute gastritis is usually initiated and promoted by *H. pylori* infection; if not eradicated, progressing into chronic active gastritis, characterized by mononuclear infiltrates and lymphoid follicles with active germinal centres, plasma cells neutrophils, eosinophils, and macrophages in the lamina propria of the gastric mucosa (Figure 3) [31].

A study performed with pediatric patients who were diagnosed with dyspepsia, both, *H. pylori*-negative and positive patients showed that intestinal microbiota is modified by *H. pylori* infection. Relative abundances of *Bacteroidaceae*, *Enterobacteriaceae*, and *Porphyromonadaceae* families and genera *Bacteroides*, *Parabacteroides*, *Streptococcus*, and *Lactococcus* were observed to be significantly increased in *H. pylori*-positive patients when compared to the microbiota of healthy subjects. *H. pylori*-positive patients also showed a significantly higher abundance of *Bacteroidaceae* and *Enterobacteriaceae*, while the families *Bifidobacteriaceae*, *Lactobacillaceae*, and *Lachnospiraceae* showed a lower relative abundance in both *H. pylori*-positive and negative patients when compared to healthy subjects [32].

Aviles-Jiménez et al. observed that all patients with non-atrophic gastritis, intestinal metaplasia, and gastric cancer (*n* = 15) shared phyla Proteobacteria and Firmicutes with a relative abundance of 70%. In this work, gradual microbiota alterations were found, specifically decreasing relative abundances of TM7, *Porphyromonas*, and *Neisseria*, while specific genera such as *Lactobacillus coleohominis*, and *Lachnospiraceae* gradually increased in the microbiota of patients with non-atrophic gastritis to intestinal metaplasia to gastric cancer, respectively [33].

Chronic infection with *H. pylori* usually results in increased heartburn, disrupting nutrient availability and the innate local response, in addition to the alterations observed in gastric physiology and immunology associated with *H. pylori* infection, e.g., the increase in atrophy and metaplasia, particularly in patients with initiation and development of gastric cancer, which have been observed to carry an altered gastric microbiota composition [34].

#### 3.3.4. Dysbiosis in Patients with Gastric Cancer and *H. pylori* Variable Status

The development of most gastric cancers involves sequential changes of the normal gastric mucosa (Figure 3A). Non-atrophic gastritis is characterized by oedema of the lamina propria, and a variable number of mononuclear cells, mainly lymphocytes, plasma cells, with or without lymph node formation; neutrophils, eosinophils, and macrophage infiltrates may also be present (Figure 3B). Atrophic gastritis (Figure 3C), in which, in addition to the changes previously described, increased distance between glands, loss of glandular units, and increased connective tissue are shown. In intestinal metaplasia (Figure 3D) the main histological change is the presence of goblet cells. The presence of dysplastic changes noted can be seen as hyperchromatic and elongation of nuclei with the loss of their normal polarity, and, finally, carcinoma [35].

Atrophic gastritis and intestinal metaplasia are well-established indicators of increased risk to develop gastric cancer [36,37,38,39,40]. The entire process is believed to be initiated and promoted by *H. pylori* [41], although it has been recently proposed that diverse microbial alterations interact in the diverse stages of gastric carcinogenesis; therefore, as we can see, dysbiosis in the stomach is considered to be a dynamic process intimately correlated with the progression to gastric cancer [42].

Castaño-Rodríguez et al. compared the gastric microbial composition of patients who were serologically *H. pylori*-positive, along with dyspepsia and gastric cancer. Identified enriched species in patients diagnosed with gastric cancer included *Lactococcus lactis*, *Fusobacterium mortiferum*, *Haemophilus parahaemolyticus*, *H. sputorum*, *Staphylococcus* spp., and *Methylobacterium adhaesivum*, while in non-nested analysis, authors identified *Veillonella atypica*, *V. dispar*, *Dialister pneumosintes*, and *Leptotrichia buccalis*. Some of these microbial communities, such as *Veillonella* and *Leptotrichia*, have been demonstrated to co-aggregate with other intestinal bacteria to produce biofilms that play a key role in other types of colorectal adenocarcinoma [43].

A study performed by Liu et al. on 276 patients with gastric cancer, assessed the diversity and composition of the gastric microbiota across three microenvironments: tumoral and peritumoral microhabitats and compared them with subjects having normal gastric mucosal morphology (healthy microenvironment). They observed that *H. pylori*, *Streptococcus anginosus*, *Prevotella melaninogenica*, *P. copri*, *Cutibacterium acnes* (formerly known as *Propionibacterium acnes*), *Bacteroides fragilis*, *B. uniformis*, *Bacillus cereus*, and *Akkermansia muciniphila* were significantly different across the three microenvironments. Interestingly, *C. acnes*, *S. anginosus*, and *P. melaninogenica* were abundantly present in tumoral microhabitats, while the presence of *B. uniformis* and *P. copri* decreased significantly; meanwhile, *A. muciniphila* and *B. fragilis* demonstrated similar bacterial alteration patterns when analysing the variation between peritumoral and tumoral microhabitats [44].

It has been recently hypothesized that bacterial species with nitrate-reducing metabolism contribute to gastric malignant transformation by crescent intragastric concentrations of nitrite and N-nitroso compounds. Ferreira et al. fully reconstituted the gastric metagenomes from patients with gastric carcinoma revealing that the functional composition of total microbiota from these patients increased the nitrate reductase functions, promoting the reduction of nitrate to nitrite, and nitrite reductase functions, promoting the reduction of nitrite to nitric oxide when compared to the functional composition of chronic gastritis patient’s microbiota. These findings provide evidence that microbial communities with genotoxic potential are present in patients with gastric carcinoma [42].

A case-control study performed by Gunathilake et al. which involved 556 participants, showed high *C. acnes*, *H. pylori*, and *P. copri* relative abundances in patients with gastric cancer when compared with the controls, whereas the relative abundance of *L. lactis* was significantly higher. Patients with these significantly higher relative abundances showed a higher risk of developing gastric cancer. Although *H. pylori* infection was observed to be the strongest single risk factor for gastric cancer, researchers also observed high *C. acnes* relative abundance in these patients, revealing that this bacterium increases the risk for the development of gastric cancer, highlighting a commensal skin bacterium recently identified as a gastric microbiota resident [45].

Eradication of *H. pylori* with multiple antibiotic therapies combined with PPIs was observed to reduce the risk of gastric cancer; an effect that may be limited to gastric cancer patients without atrophy and metaplasia [46,47,48]. In patients with malignant progression of gastric cancer, *H. pylori* eradication was observed to reduce symptomatology, but did not stop the progression of the neoplasia, indicating that cancer can occur even ten years after *H. pylori* eradication treatment [49].

#### 3.3.5. Alterations in the Gastric Microbiota after Multiple Therapeutic Interventions

Self-medication is considered a potential contributor to human pathogen resistance to antibiotics. However, adverse consequences of such practices are difficult to evaluate due to affected bacteria by some aggressive drugs (e.g., multiple antibiotic therapies, proton pump inhibitors) are just not pathogens, but also gastrointestinal commensal bacteria, altering the native microbiota.

#### 3.3.6. Abuse of Proton Pump Inhibitors

The use of PPIs is common in patients diagnosed with gastroesophageal reflux and functional dyspepsia. PPIs are defined as prodrugs that are activated by acid and lower pH of the local microenvironment. PPIs are known as weak bases (due to their pK_a_ ≤ 4). These compounds are rapidly activated by the acids previously secreted by gastric parietal cells. High acidity acts as a protonation factor for activation of these weak bases to form disulphides (thiophilic drugs) by binding to cysteine residues residing in the (H+/K+ ATPase) proton pumps. Disulphides react with luminally accessed cysteines on the acid pump enzyme, blocking acid transport and altering gastric acidity to pH = 7 [50].

The microenvironmental changes generated by PPIs alter the native gastric conditions significantly and develop histopathological damage including the enlargement of fundic glands (Figure 3E,F) and modifying the commensal microbiota composition. Parsons et al. compared the microbiota profiles in different patient groups treated with PPIs: patients diagnosed with autoimmune atrophic gastritis, patients with *H. pylori*-induced gastritis and atrophy, and healthy controls. Patients who received PPIs showed similar bacterial profiles to those found in the stomachs of healthy subjects, despite high levels of serum gastrin concentrations. Alpha indexes showed samples from patients treated with PPIs were less diverse than normal gastric samples. Evaluation of the evenness index showed that bacterial communities from PPI-treated samples and normal gastric samples were both equal in relative abundance. Richness calculations demonstrated that normal gastric samples showed the highest diversity when compared to all the other groups, while the *H. pylori*-infected groups (both, *H. pylori*-induced atrophy and gastritis) presented with a significantly fewer number of species. In PPI-treated patients, *Streptococcaceae* (17%) was the most abundant family, outranking *Prevotellaceae* (11%), *Campylobacteraceae* (5%), and *Leptotrichiaceae* (4%). At the operational taxonomic unit (OTU) level, *Cyanobacteria* and *Streptococcus* relative abundances were significantly increased in PPI-treated patients; however, the relative abundances of genera *Actinobacillus*, *Porphyromonas*, *Leptotrichiaceae*, *Prevotella*, *Treponema*, *Haemophilus*, *Fusobacterium*, and *Tannerella* were significantly decreased in patients treated with PPIs [51].

In a second study performed on 12 patients who presented dyspepsia and were treated with a PPI, the phyla Proteobacteria, Actinobacteria, Fusobacteria, Bacteroidetes, and Firmicutes represented the five most abundant phyla in 98% of total sequence reads. Among them, Proteobacteria, Bacteroidetes, and Firmicutes were dominantly abundant; 115 genera were identified in total, including the genera *Helicobacter*, *Prevotella*, *Streptococcus*, *Veillonella*, *Neisseria*, *Porphyromonas*, *Fusobacterium*, *Gemella*, *Haemophilus*, and *Leptotrichia*, representing the ten most abundant genera (in decreasing order, respectively) [52].

#### 3.3.7. Use of Antibiotics

Successful bacterial eradication treatments significantly affect the gastrointestinal microbiota [23]. Increased improvements in sanitization, hygienical measures, and the extensive use and abuse of antibiotics are considered the most important factors responsible for the reduction in infectious disease transmission, making it important in the induction of significant alterations in the native microbiota composition [2,53]. The consequences of antibacterial therapies are difficult to evaluate since the affected bacteria not only includes pathogens but also beneficial bacteria useful for maintaining human health [2].

Dysbiosis is the most important consequence of adverse effects of antibiotic therapies, which has favored the presence of microorganisms carrying antibiotic resistance genes, facilitating horizontal gene transfer between commensal and pathogen bacteria, as well as assisting in the recolonization of gastrointestinal mucosa by pathobionts [54]. Antibiotic properties, such as spectrum, dose and duration of administration, pharmacokinetics and pharmacodynamics, and the route of administration contribute to modifying the human microbiota [55].

#### 3.3.8. *H. pylori* Eradication Treatments

*H. pylori* infection induces a mild-to-severe inflammatory process; however, the gastric mucosa of patients with different gastric alterations can harbor non-*H. pylori* bacteria, which can induce and potentialize inflammatory processes that can lead to a malignant transformation in the stomach, e.g., genera *Acinetobacter* and *Klebsiella*, associated with gastric carcinogenic processes [56,57]; genus *Cutibacterium*, which is a dominant genus in pilosebaceous follicles and a native member of mucosa and skin microbiota, was recently named a pathobiont related to the microbiota of immunocompromised patients and considered as a trigger of corpus-dominant lymphocytic gastritis [58,59,60]; and genus *Corynebacterium*, as a member of human native microbiota, is frequently isolated from the skin, mucosal membranes, and gastrointestinal tract [61,62,63].

For more than 30 years, it has been observed that multiple antibiotic therapies, which usually include metronidazole, mainly affect anaerobic and microaerophilic microorganisms. The metronidazole antimicrobial spectrum activity includes anaerobic bacteria, such as *Peptococcus*, *Eubacterium*, *Clostridium difficile*, *C. perfringens*, *Veillonella*, *Bacteroides fragilis*, *Fusobacterium*, and *Peptostreptococcus* [64]. However, when administering multiple antibiotic combination therapies with a PPI, dysbiotic states are generated in the whole gastric microbiota. In Table 1, the principal alterations to the gastric microbiota after the eradication of *H. pylori* infection with several therapies (i.e., standard triple therapy or quadruple therapy) in patients with distinct diseases are shown. As can be seen, not only microaerophilic or anaerobic bacteria are affected by these therapies, but also facultative and aerobic bacteria, although in different relative abundances and richness parameters. This observation may be attributed to the interindividual variability of the study subjects, which usually makes it difficult to identify the distinctive pathogenic species among patients at risk of developing gastric diseases from those who are not at risk [20,65]. Li et al. reported the effect of *H. pylori* eradication on the gastrointestinal microbiota of patients with duodenal ulcers. Before the eradication treatment, the *Clostridium leptum* and *Prevotella* subgroups were aberrantly different between gastric corpus and antrum. Pre-treatment peptic ulcer patients who were administered with antibiotic therapies (i.e., rabeprazole, colloidal bismuth, amoxicillin, and clarithromycin) for *H. pylori* eradication demonstrated that the relative abundances of the *Lactobacillus* group, *Enterobacteria*, and *C. leptum* subgroup were significantly increased in antrum, while for the *Clostridium coccoides* subgroup relative abundances were lower. After treatment, recolonization with *Lactobacillus* was more abundant in antrum than in the corpus. *H. pylori*-negative patients with antral gastritis showed higher *Enterobacteria* relative abundances, suggesting that *H. pylori* inhibited the distribution of these bacterial families [66]. 

*H. pylori* eradication can result in resilience, which is defined as the microbial composition recovery whether by growth, physiological or genetic adaptation, after composition alterations due to the host, or environmental factors of the gastric microbiota. Gastric resilience can occur with specific bacterial species such as *Granulicatella*, *Streptococcus*, *Rothia*, *Leptotrichia*, *Acinetobacter*, *Faecalibacterium*, *Rahnella*, *Kaistobacter*, *Blautia*, *Caulobacter*, *Nocardioides*, and *Brevundimonas*. However, recolonization and the increase in relative abundances with these bacterial species can be associated with the emergence of atrophy or intestinal metaplasia (IM) following one year of *H. pylori* eradication (e.g., *Granulicatella*, *Actinomyces*, *Rothia*, *Peptostreptococcus*, *Abiotrophia*, and *Parvinomas*), or the presence of persistent inflammation processes (e.g., *Acinetobacter lwolffii*, *Streptococcus anginosus*, *Ralstonia*, *Erwinia*, and *Prevotella*) [71].

#### 3.3.9. Microbiota Recovery after Antibiotic Eradication Treatment

Antibiotic therapies (monotherapies or multiple antibiotic combinations) are one of the most extreme disturbances occurring in the human microbiome [72]. Therefore, increased focus is being given to evaluate how the gastrointestinal microbiome is impacted by antibiotic use, both by acute and long-term treatments [55], rendering individuals more susceptible to infections [21].

The search for new alternatives in antimicrobial therapies for pathogen eradication is much needed, with a special interest in natural product-based therapies [73], such as faecal microbiota transplantation (FMT), and supplementation of probiotics.

Defined as the transfer of stool from a healthy donor into the gastrointestinal tract of a patient to restore the composition of the microbiota [74], faecal microbiota transplantation (FMT) represents a promising paradigm for treating conditions where the microbiome and organ dysbiosis contributes to pathophysiology [75]. Although FTM is almost recognized as a safe therapeutic protocol, adverse effects can be developed due to short post-FTM follow-ups [74]. FTM can lead to the induction of several diseases, such as obesity, IBD, autism, asthma, among others. On the other hand, specific bacterial overgrowth of some species (e.g., *Enterococcus faecalis*, *Bacteroides fragilis*, and *Escherichia coli*) may increase the risk of developing cancer. Rare cases have been documented, where FTM-associated bacteria can cause serious problems, or even death [74], suggesting the employment of alternative therapies for optimal recolonization, such as probiotics.

#### 3.3.10. Probiotics

Probiotic supplementation therapies are emerging therapies for *H. pylori* treatment [76]. Probiotics are defined as live bacteria that usually coexist in symbiosis within the human host. They are believed to improve human health when consumed or applied [73]. Lactic acid bacteria (LAB) have been usually employed as probiotics. Lactobacilli, bifidobacteria, and other LABs are isolated from fermented dairy products and the faecal microbiota. When administered, they usually interact with both the host and established microbiome through molecular effectors which are present on the bacterial cell structure or secreted as metabolic products, such as butyrate compounds, secreted proteins, such as extracellular proteins, organic acids, indole, bacteriocins, H_2_O_2_, and NO molecules [67,77]. These secreted products generate a reaction in the microbiota by cross-feeding interactions, alterations in the microenvironment, competition for nutrients and specific binding sites, and growth inhibition of other microbes by the production of bacteriocins. The effects of probiotics to mediate health benefits on the microbiota contribute to the inhibition of pathogen overgrowth states. The direct interaction between probiotics and receptors in the gastrointestinal epithelium produces important effects such as the enhancement of intestinal barrier integrity and prevention of inflammation, as well as systemic effects via host, immune, endocrine, and nervous system mediators [77].

In vitro experiments have demonstrated that several probiotics have the potential to antagonize *H. pylori* through metabolite production, secretion, or bacterial cells. Several studies have determined the capability of four strains of *Lactobacillus* for inhibiting *H. pylori* growth [78], urease activity suppression [79], drug-sensitivity, and drug-resistance through the secretion of lactic acid by *L. pentosus* LPS16 [80]; bacteriocin production and secretion can generate oxidative damage to membrane lipids, pathogenic proteins, and DNA by the formation of peroxide ions [81], such as those secreted by *L. bulgaricus*, that can inhibit not only antibiotic-sensitive *H. pylori* strains but also inhibit antibiotic-resistant strains [82]. Moreover, the effect of drug synergy, mutant prevention, biofilm, and gastrointestinal tract microbiota on *H. pylori* growth inhibition for maintenance of both gastric and intestinal microbiota has also been demonstrated [76].

The recent development of low-cost whole-genome sequencing has allowed the characterization of new bacteria with high potential for health benefits, with the opportunity to be developed as probiotics [77]. Although beneficial effects and mechanisms of probiotics in the gastrointestinal tract have been demonstrated [21], there is evidence that probiotics may also act in the treatment and prevention of infectious diseases. Recently, bacterial candidates have been isolated from the human gut that showcased probiotic potential (e.g., *Roseburia intestinalis*, *Faecalibacterium prausnitzii*, *Eubacterium* spp., *Bacteroides* spp., *Akkermansia municiphila*), thus expanding the possibilities of research on probiotics and their relationship with emerging healthcare challenges [77].

## 4. Conclusions

During the development of gastric diseases and their therapeutic treatments, the native microbial composition of the patient can be altered, generating dysbiotic states. The presence of *Helicobacter pylori* usually induces dysbiosis in the stomach, in some cases defining serious diseases, e.g., gastric cancer. Its eradication with different therapies causes significant alterations that might not always carry optimal colonization, e.g., metabolic and immunological implications, or even the initiation and development of gastric disorders caused by non-*H. pylori* bacteria. The use of alternative antibiotic therapies, such as probiotics, can improve, in most cases, the process of bacterial resilience.

## Figures and Tables

**Table 1:** Dominant native microbiota and alterations in gastric microbiota after *Helicobacter pylori* (HP) eradication.

Study Subjects	Dominant Native Microbiota	Treatment	Microbial Alterations	Reference
*H. pylori* (HP) dyspeptic patients (Culture-dependent methodology)	↑*S. salivarius*, *S. mitior*, *S. sanguis*, *Micrococcus*, *Peptostreptococcus*, *Lactobacillus*, *Bifidobacterium*, *Fusobacterium*, *Veillonella*	Omeprazole (20 mg) Amoxicillin (1 g) Metronidazole (400 mg)	↑*S. intermedius*, *Neisseria*	[67]
	↓*Staphylococcus*, *S. intermedius*, *Haemophilus*, *Neisseria*, *Enterobacteriaceae*, *Prevotella*, *Bacteroides*		↓*S. salivarius*, *S. mitior*, *S. sanguis*, *Staphylococcus*, *Micrococcus*, *Peptostreptococcus*, *Lactobacillus*, *Bifidobacterium*, *Veillonella*, *Prevotella*, *Bacteroides*, *Fusobacterium*	
	↑*S. salivarius*, *S. mitior*, *Enterobacteriaceae*, *Peptostreptococcus*, *Lactobacillus*, *Bifidobacterium*, *Veillonella*, *Prevotella*, *Bacteroides*, *Fusobacterium*	Omeprazole (20 mg) Clarithromycin (250 mg) Metronidazole (400 mg)	↑*Staphylococcus*, *Micrococcus*, *Enterobacteriaceae*, *Veillonella*	
	↓*S. intermedius*, *S. sanguis*, *Staphylococcus*, *Micrococcus*, *Haemophilus*, *Neisseria*		↓*S. salivarus*, *S. mitior*, *Peptostreptococcus*, *Lactobacillus*, *Bifidobacterium*, *Prevotella*, *Bacteroides*	
HP peptic ulcer (Real-Time PCR)	↑*Prevotella*, *C. leptum* ↓*Enterobacteria*	Rabeprazole (20 mg) Colloidal Bismuth pectin Clarithromycin (500 mg) Amoxicillin (1 g)	↑*Lactobacillus*, *C. leptum*, *Enterobacteria* ↓*C. coccoides*	[68]
HP chronic gastritis	↑*H. pylori* (83.70%), *Eubacterium*	Esomeprazole (20 mg) Amoxicillin (1 g) Clarithromycin (500 mg)	↓*H. pylori* (6.88%)	[69]
HP intestinal metaplasia	↑Proteobacteria no-HP (4.55%)		↑Bacteroidetes, Fusobacteria, Actinobacteria	
HP-negative patients	*Haemophilus*, *Serratia*, *Neisseria*, *Stenotrophomonas*		↑Proteobacteria no-HP (51.70%)	
HP gastric cancer (NGS)	↑Proteobacteria *Flavobacterium*, *Klebsiella*, *Serratia*, *Stenotrophomonas*, *Achromobacter*, *Pseudomonas*		*Flavobacterium*, *Neisseria*, *Serratia*, *Fusobacterium*	
HP-positive and negative patients (NGS)	*Bacteroidetes*:*Firmicutes* (0.94:0.84) *Bifidobacterium*, *Lactobacillus*, *C. butyricum*, *Faecalibacterium prausnitzii*, *Akkermansia municiphila*	Pantoprazole (40 mg) Amoxicillin (1 g) Furazolidone (100 mg) Colloidal Bismuth pectin (400 mg)	↑Proteobacteria, Cyanobacteria ↓Firmicutes, Bacteroidetes, Verrucomicrobia, Lentisphaerae ↓*Ruminococcaceae*, *Lachonspiracea*	[48]
HP-negative patients (NGS)	*Nitrospirae*		↑*Enterobacteriaceae*, *Leuconostococaceae* ↓*Rikenellaceae*, *Christensenellaceae*, *Peptococcaceae*, Clostridiales Family XI, *Victivallaceae*	
HP-positive patients with different gastric pathologies (NGS)	↑*Eubacterium*, *Bacteroides*, *Prevotella*	Amoxicillin (1 g) Clarithromycin (500 mg) Bismuth Subsalicylate (240 mg) Esomeprazole/Panteprazole (20 mg)	↑Betaproteobacteria, Gammaproteobacteria *Bacteroides*, *E. faecium* ↑*Enterobacteriaceae*, *Siphoviriadae* ↓Actinobacteria, Verrucomicrobia, Synergistia ↓*B. adolescentis Bifidobacteraceae*, *Coriobacteriaceae*, *Eubacteriaceae*, *Lachnospiraceae*, *Ruminococcaceae*	[70]
HP-positive patients associated to:		Omeprazole (20 mg) Amoxicillin (1 g) Clarithromycin (500 mg)		[66]
(1) Atrophy	↑*Moraxella*, *Pasteurella*, *Bulleidia*, *Agrobacterium*		↑*Pseudomonadaceae*, *Oxalobacteraceae*, *Microbacteriaceae*, *Enterobacteriaceae*, *Lachnospiraceae*, *Vibronaceae*, *Halomonadaceae* ↑*Acinetobacter*, *Ralstonia*, *Actinobacillus*, *Erwinia, Granulicatella*, *Streptococcus*, *Rothia*, *Leptotrichia* ↓*Helicobacter*, *Sphingomonas*, *Roseburia*, *Haemophilus influenzae*, *Actinobacillus parahaemolyticus*, *Neisseria subflava*	
(2) Intestinal metaplasia at progression	↑*Pseudomonas*, *Peptostreptococcus*, *Parvimonas*, *Halomonas*		↑*Peptostreptococcus*	
(3) Intestinal metaplasia at regression (NGS)	↑*Lachnospira*, *Kaistobacter*, *Campylobacter*, *Devosia*, *Sphingobium*		↑*Peptostreptococcus*	

↑: Increase of relative abundances; ↓: Decrease of relative abundances; NGS: Next-Generation Sequencing.

## Data Availability

The data presented in this study are openly available.
